# Modeling walking accessibility to urban parks using Google Maps crowdsourcing database in the high-density urban environments of Hong Kong

**DOI:** 10.1038/s41598-023-48340-w

**Published:** 2023-11-27

**Authors:** Fang-Ying Gong

**Affiliations:** 1 School of Public Administration and Policy, Renmin University of China, Beijing, China; 2 School of Architecture, The Chinese University of Hong Kong, Hong Kong, China

**Keywords:** Psychology and behaviour, Sustainability

## Abstract

Accessing urban parks is important for promoting physical activities and improving public health. In this study, we propose the use of Google Maps crowdsourcing data and the incorporation of park attractiveness to model urban park accessibility in the complex urban environments of Hong Kong. The difference between using geometric and route distance, the effect of park attractiveness in measuring accessibility, and the benefits gained from using walk time compared to distance are investigated. Our result shows that (1) route and geometric distances have a strong correlation with a conversion factor of about 1.5; (2) the common assumption that park size can be a proxy for describing attractiveness may not be correct. Instead, park attractiveness should be explicitly considered for a more effective accessibility modeling; and (3) estimation by walking time shows that there are non-negligible impacts from street conditions and traffic on urban park accessibility. Moreover, district hotspots short of park accessibility or attractiveness can be explicitly detected. Overall, this developed approach provides a flexible and informative approach to model the accessibility to urban parks. The outputs will help city planners, health professionals, and policymakers to evaluate and improve urban park planning and equity in accessibility in high-density cities.

## Introduction

Access to urban green spaces, including urban parks, has contributed to increased physical activities, public health advancement, and socialization of urban residents^1,2^. As an important component of public facilities, urban parks are important for improving the quality of life in high-density cities, such as Hong Kong, by providing public spaces for recreation, physical exercise, interaction with nature, and social activities that can promote both personal health and social cohesion within communities^3^. Increasing accessibility to nearby green spaces including parks has been shown to be positively associated with improving residents’ health^4^. However, existing public open spaces and areas in Hong Kong have inequitable geographical distribution^5^. This inequitable distribution may deprive underprivileged communities in high-density mass housing zones of the right to conveniently access public space. It is crucial to evaluate the accessibility of open spaces including urban parks in Hong Kong, for the purpose of quantifying their provisions for urban residents and providing science-based evidence for city planners to improve current open space planning and design in high-density cities.

Methods developed by previous studies for measuring spatial accessibility of neighborhood parks can be grouped into three general approaches, including (1) spatial proximity approach, which simply measures travel costs using distance or time from residents to parks (e.g., ref.^6^), (2) container approach, which measures the number or total area of parks within a defined geographic region (e.g., ref.^7^), and (3) gravity model-based approach, which models the spatial interaction between residents and parks based on the travel distance and park provisions (e.g., ref.^8,9^). Based on the classical gravity model, Zhang et al.^10^ developed a new measure of spatial access to parks, the population-weighted distance (PWD), which combines the advantages of current park access approaches and incorporates the information processing theory and probability access surface model to more accurately quantify residential population’s potential access to parks. Based on the above methods, an increasing number of studies has quantified the accessibility to public open spaces to understand the disparities and inequalities^7,11–13^. However, results from these analyses have diverged considerably depending on the methods used, which have different parameters to model accessibility. Moreover, these methods lack a comprehensive modeling of resident-park interactions, in which the full dimensions of park characteristics (e.g., proximity, size, and quality) and street and traffic conditions for pedestrian should be explicitly considered.

Previous studies have shown evidence that urban residents prefer proximate, larger, and attractive public open spaces (e.g., ref.^14^), which correspond to the three most important parameters in modeling accessibility: (1) park proximity by travel distance or time, (2) park size, and (3) park attractiveness determined by its quality^3,10,14,15^. However, the contributions of these factors in modeling accessibility of urban parks have not been well quantified, including (1) the impact of using geometric distance, for simplicity, in measuring accessibility in the context of a high-density city; (2) the relationship between park size and attractiveness, and the effect of incorporating park attractiveness in measuring urban park accessibility; and (3) the difference between using travel-time and -distance in measuring spatial proximity between residents and urban parks.

Street and traffic conditions, such as road types and street lights, influence pedestrian’s walking speed and have significant impact on urban park accessibility, but are often ignored due to the difficulty in quantifying these parameters. Crowdsourcing data from the widely used Google Maps, which provides estimates of optimized walking distance and time based on massive historical user big data, offer a unique data source that contain real life street and traffic information at different times in a day (e.g., peak hour and off-peak hour). Similar web map services are also available from Baidu and Gaode in China^16^. For calculating route walking distance and time, these web map services are utilized by using its Application Programming Interface (API). These web maps data have been used and analyzed in several previous studies^16–26^ and shown to be consistent with independent data source^27^. In particular, using Google Map APIs, Gu et al.^17^ estimated the travelling distance and time to optimize the locations of the preventive health care facility; Kobayashi et al.^18^ developed a geographical information system to provide guidance to the nearby referral hospitals; Xia et al.^22^ obtained dynamic estimation of travel time across cities in Australia. Similar studies have been carried out using Gaode web map API services. For example, Rong et al.^24^ used Gaode web map API service to estimate the travel time and evaluate the spatial equity of medical facilities in Zhengzhou, China; Zhang et al.^16^ developed a green accessibility index to quantify the accessibility of public green spaces in urban areas. The above-mentioned studies have demonstrated the capability of the existing web map APIs in providing accurate and dynamic estimate of travel distance and time. However, few studies have been conducted to incorporate the web map API data in evaluating urban park accessibility in a high-density urban environment such as downtown Hong Kong.

In this study, we propose the use of Google Maps crowdsoucing database and the incorporation of park attractiveness to model the urban park accessibility. Since realistic traffic and road conditions, which are often very complex in high-density environments, are reflected in the Google Maps data, the approach is supposed to be suitable for complex urban environments like Hong Kong. Pedestrian access based on walking, the most common way to access urban parks by the elderly and children^28^, is examined in this study. From comparison of different modeling methods, we investigate the benefits of the proposed approach from the following three aspects: (1) using the route distance from Google Maps API, which is more realistic than the traditional geometric distance; (2) incorporating park attractiveness quantified using its attributes, facilities, and amenities; (3) using travel-time instead of -distance to measure spatial proximity between residents and urban parks. Given the complexity of urban settings and landscapes in the high-density city of Hong Kong, travel distance may not be proportional to travel time. A comparison between time and distance will shed light on how the urban street conditions such as street slopes and traffic may affect urban park accessibility. These results can provide scientific evidence for future design and planning of urban space in Hong Kong and similar high-density urban areas in the world.

This paper is structured in the following framework. Used data and methods for modeling accessibility are described in Section "Methods". Results of accessibility modeling and the associated analysis are given in Section "Results". Further discussion is presented in Section "Discussion", followed by conclusions in Section "Conclusions".

## Methods

### Study area

Hong Kong is a typical high-rise and high-density city. It has a total population of 7.24 million (mid-2014) living in a compact area of 1104 km^2^ with high population density of about 6690 persons per square kilometers on average , which makes Hong Kong one of the most densely populated cities in the world. Strictly limited by urban policies for land use planning, the sprawl of built-up areas, new towns and metropolitan area (272 km^2^) account for only about 25% of the total areas. The average per capita public open space provision in Hong Kong is 2.46 m^2^/person^29^, which is lower compared with other Asian cities such as Macau peninsula (2.7m^2^/person)^30^, Singapore (10 m^2^/person)^31^, and Tokyo (7 m^2^/person)^32^. Moreover, local open space per capita in four populated districts, including Central and Western, Wanchai, Yau Tsim Mong and Kowloon City districts is even below 1 m^2^/person^33^. As a result of high population and building density but very limited open spaces, a high degree of fragmentation, shortage of green space, and weak accessibility and connectivity are the major problems of green spaces planning in Hong Kong^34–36^.

**Figure 1:**
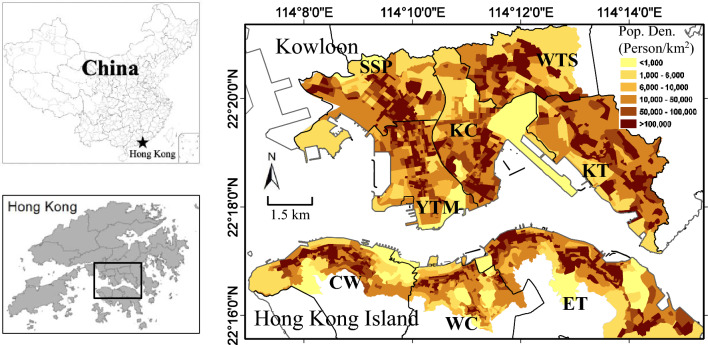
Location of Hong Kong (left panel) and its high-density urban areas (right panel) including eight district councils (labeled in short names) with five in Kowloon: Kowloon City (KC), Yau Tsim Mong (YTM), Sham Shui Po (SSP), Kwun Tong (KT), and Wong Tai Sin (WTS), and three in Hong Kong Island: Central & Western (CW), Wan Chai (WC), and Eastern (ET). The population density shown in the right panel is based on the street block level. The statistics of demographic are publicly available from the 2011 census in Hong Kong. The maps are created using ArcGIS software (version 10; https://www.esri.com/).

In this study, the high-density District Councils (DCs; the second level of planning unit), where the population density is over 6000 persons/km^2^, of Hong Kong Island and Kowloon are selected as the study area, as shown in Fig. 1. In total, 8 DCs are selected, including 5 districts in Kowloon area (Kowloon City, Yau Tsim Mong, Sham Shui Po, Kwun Tong, and Wong Tai Sin) and 3 districts in Hong Kong Island (Central & Western, Wan Chai, and Eastern). The study area can also be sub-divided into 104 Tertiary Planning Units (TPUs; the third level of planning unit) and 2243 Street Blocks (SBs; the fourth level of planning unit), respectively. Figure 1 shows the population densities in SB level. The extremely dense population (> 100,000 persons/km^2^) are distributed in all 8 DCs and a cluster exists in West Kowloon (including Sham Shui Po and Yau Tsim Mong). As a high-density city as well as a rapidly aging society with very limited public open space, easy access to urban parks is important in promoting physical and social activities and improving public health, especially for elderly, in Hong Kong^5^. Therefore, quantification and assessment of urban park provisions are essential for providing science-based evidence in urban planning and design strategies for the equitable provision of urban green spaces in Hong Kong.

### Data

#### Urban parks in Hong Kong

**Figure 2:**
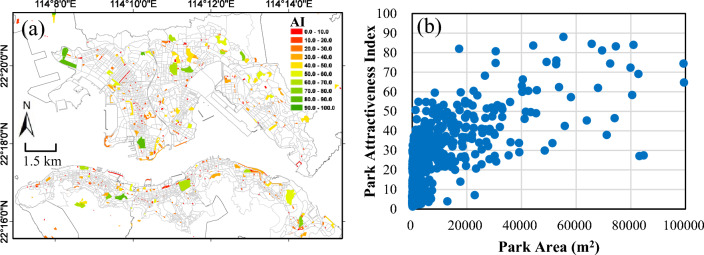
(**a**) The distribution of 902 urban parks (color shaded according to the Attractiveness Index (AI) for each park described in Section "Attractiveness index (AI) for urban parks") and the 2243 street blocks (in dashed thin line) in the study area; The map is created using ArcGIS software (version 10; https://www.esri.com/). (**b**) Correlation between park sizes and the corresponding AIs from all urban parks in the study area (R^2^ = 0.19). For the purpose of showing more details, parks with the area over 100,000 m^2^ (13 of them) are not shown.

This study focuses on the publicly accessible urban parks, including mini-park (for unique recreational needs), neighborhood park (for recreational and social activities of the neighborhood), community park (for community-based recreation needs), and large urban park (for broader purposes of community- and neighborhood-based recreation needs as well as preserving unique landscapes)^37^, which are the main category of public open space in high-density urban areas of Hong Kong^5^. The spatial distribution of urban parks used in this study is derived from the urban facility layer of the B5000 maps series (with a spatial accuracy of 15 to 150 cm) from the Hong Kong Survey and Mapping Office at the Lands Department^38^. All 902 patches of urban parks with different sizes are used in this study since even small size parks can have a non-negligible impact^3^. These parks are scattered in the study area (Fig. 2a), with mean park size of about 10,052 m^2^, and maximum and minimum area size of about 10 m^2^ and 687,237 m^2^, respectively. The details of these urban parks are shown in Table 1. The DCs in Kowloon generally have larger urban park area ratio than those in Hong Kong Island even the numbers of urban parks are comparable since the parks in Kowloon have larger areas. In this study, the spatial accessibility to public urban parks is calculated at the SB level and further aggregated to TPU level. The centroid of each street block is used as a point of origin to measure the distance to each public park.

**Table 1:** Basic statistics of the public urban parks in high-density urban areas of Hong Kong.

	Park number	Minimum area (m^2^)	Maximum area (m^2^)	Mean (m^2^)	Area ratio (%)
Hong Kong Island					
Central&Western	68	75	69,504	6106	3.28
Wan Chai	52	78	9204	1783	0.98
Eastern	85	25	174,237	8941	4.07
Kowloon					
Yau Tsim Mong	66	111	117,337	6564	6.15
Sham Shui Po	38	263	1,210,834	10,540	4.30
Kowloon City	46	324	99,460	9566	4.23
Wong Tai Sin	31	264	80,512	12,284	4.09
Kwun Tong	53	161	63,924	7212	3.55

#### Walking distance and time estimations from Google Maps API

**Figure 3:**
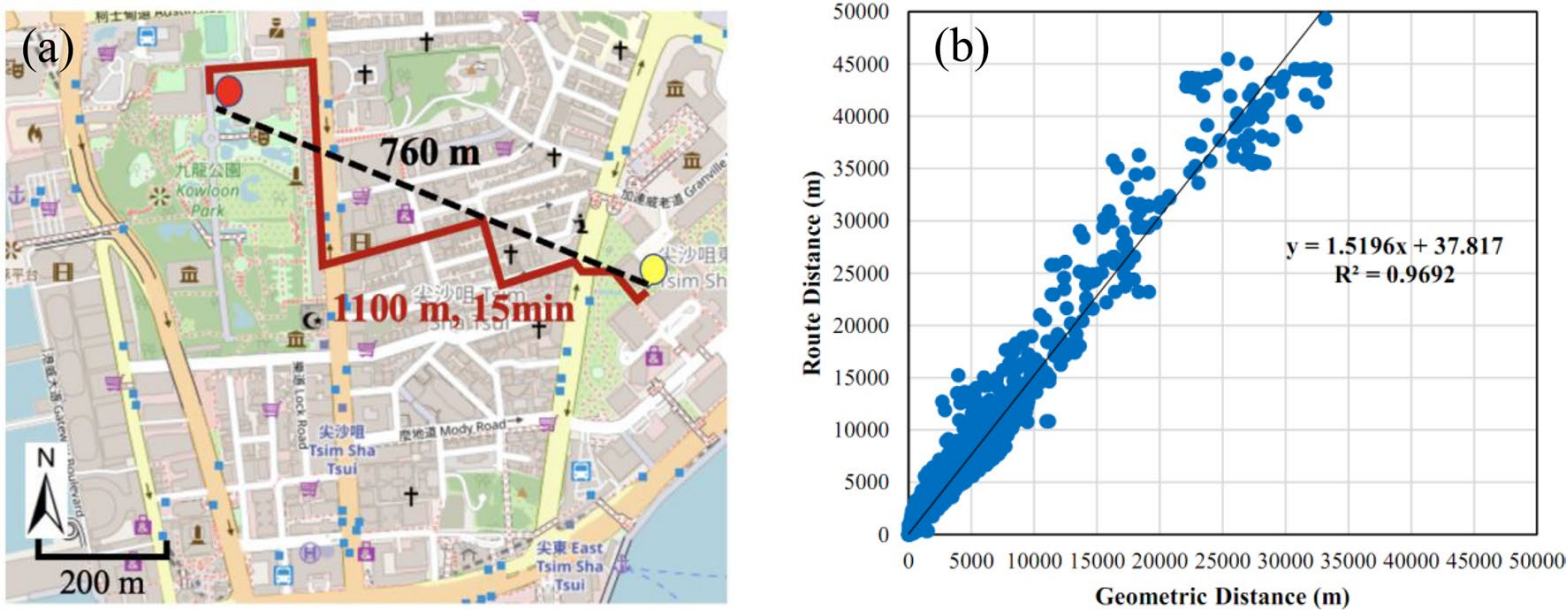
(**a**) Example of geometric (black dash) and route (red solid) walking distance from a residential point (yellow) to an urban park destination (red) calculated using Google Maps Distance Matrix API. The route walking distance and time are also indicated. The base map is from Open Street Map (www.openstreetmap.org). (**b**) Scatter plot and linear regression of route and geometric distances from all SB units to the nearest urban parks. The estimated formula for linear regression is also indicated. The two distance measures have a high correlation with R^2^ of 0.97. On average, the ratio of the route to geometric distance is about 1.52 in high-density urban areas of Hong Kong.

Distance is a key indicator of spatial accessibility to urban parks. Two common distance metrics, including route distance and geometric distance, have been widely used in the literature to study the accessibility to urban open spaces and services (e.g., ref.^6,11^). Route distance is a measure of the shortest length of a street network linking residents and destination urban parks, while geometric distance is the length of the straight geometric line between two locations based on their latitudes and longitudes, as shown in Fig. 3a. For practical reasons, geometric distance has been widely adopted because of its simplicity, even though, in theory, it is always smaller than the more realistic route distance^39^, which, however, requires the detail urban route network. Compared with travel distance, travel time is a less considered indicator when measuring the urban park accessibility given the difficulty in measuring time. However, travel time can be a more important consideration than distance for residents to visit an urban park when road conditions, such as traffic, road slopes and elevation steps which may greatly reduce people’s walking speed. Especially in Hong Kong where road conditions within the urban areas are complex because of heavy daily traffic and large variations in street elevations. In this study, urban park accessibilities in terms of walking distance and time are modeled and analyzed.

Latitudes and longitudes of the centroids of SBs and urban parks are used to calculate geometric distance based on great-circle distance. For calculating route walking distance and time, the route network data in Hong Kong based on Google Maps Distance Matrix (GMDM) are utilized by using its Application Programming Interface (API)^40–42^. The GMDM API is a service that provides travel distance and time for an origin and destination. The route search algorithm used by GMDM is to find the optimal route and best estimate of time given what is known about both historical road conditions and live traffic based on historical walking and traffic condition data collected by Google Maps^40,43^. To utilize the API for query, latitude and longitude coordinates of origins and destinations are required. Specifically, a JavaScript code with key information of latitude and longitude for the origin and the destination, travel mode, and departure time is passing to the Distance Matrix API. The travel mode of walking is selected. To be consistent, the walking distance and time used in this study are requested from GMDM in the morning between 8:00 am to 10:00 am when there are high-frequency visits to urban parks, according to a survey conducted in Hong Kong^44^. The API returned results of the detailed route files and corresponding travel costs including time and distance. In this study, the combination of origins (2,243 street blocks) and destinations (902 urban parks) results in 2,023,186 API queries.

To better understand the correlation between geometric and route distance, we correlate all the measured distances from SBs to urban parks using linear regression, as shown in Fig. 3b. The geometric and route distances show a significant linear relationship, with a coefficient of determination (R^2^) of about 0.97. This strong linear correlation agrees with previous studies (e.g., ref.^39^). Interestingly, route and geometric distances have a conversion factor of about 1.52, which suggests previous studies using geometric distance for its simplicity may have underestimated the accessibility metric by about 1.52 times.

#### Attractiveness index (AI) for urban parks

Park attractiveness is an important park attribute relevant to improving residents’ physical activity levels by attracting visitors willing to travel a longer distance beyond their neighborhood urban parks. Moreover, even in small public open spaces of equivalent size, open spaces with more attributes attract more users^14^. In this study, we quantify the park attractiveness by establishing an Attractiveness Index (AI) for each urban park. The AI is estimated based on the characteristics of amenities and facilities in a park^14,42^. The AI is quantified based on a composite score derived from ten weighted items in three categories (sports and recreation, nature, and general amenities) as shown in Table 2. These three categories are adopted from the Desktop Auditing Public Open Space Tool^45,46^ with sub-categories. Pets permission is not considered here given that it is much less important compared to the other three other categories in urban parks in Hong Kong. Also, running dogs or others pets are forbidden in the parks of Hong Kong, according to Wong^44^. The sub-categories include activity spaces and children’s playground for “Sports and Recreation”; Water features, wildlife and gardens, trees, and walking path for “Nature”; Public art, public toilets, car parking and barbeque facilities for “General Amenities”. We use normalized difference vegetation index (NDVI) as a proxy for the quantity of greenery inside a park. Facilities and amenities in parks are extracted from the urban facility layer of the B5000 maps series. The availability of park lights is not considered here since a majority of urban park visiting and physical activities happen in the daytime^44^. The attractiveness of different category should be different given its different functions provided to urban residents and therefore may not be equally important. Based on an environmental audit and personal interviews, Giles-Corti et al.^14^ assigned weights to park attributes based on the importance of each attribute to participation in physical activity from an expert panel. We re-organized these weighting values according to the three categories in this study, as shown in Table 2. For simplicity, the weighting value for each category is equally divided for the attributes inside each category. The AI score for i^th^ urban park (${AI}_{i}$) is given by,

1 $$AI_{i} = \sum BI_{j} {*}w_{j}$$

where ${BI}_{j}$ is a binary indicator of 0 or 1 given the presence of the j-th attribute, and ${w}_{j}$ is the weighting value for the j-th attribute. The AIs for urban parks in the study area are shown in Fig. 2a. As expected, larger parks in general show higher AIs, a common assumption made by previous studies (e.g., ref.^10,11^), because larger parks usually provide more walking routes and often have more facilities for entertainment and exercise^47^. To gain more understandings on the relationship between park size and its attractiveness, Fig. 2b shows the scatter plot of AIs and the sizes of all urban parks in the study area. We can see that, in general, AI increases as the park becomes larger. However, the correlation has very large uncertainty (R^2^ = 0.19). For example, for different parks with the same area, their attraction varies greatly. On the other hand, some parks with different areas show similar AIs. This comparison suggests that the common assumption that park size can be a proxy for quantifying its attractiveness is not necessarily feasible in Hong Kong. To quantify the difference, this study compares results from accessibility models by adding and not adding AIs, for the purpose of obtaining a more reasonable and effective measurement of resident-park interactions.

**Table 2:** Urban parks attributes and their corresponding weights for calculating Attractiveness Index (AI) for a urban park.

Attributes	Contents	Data source	Weight assigned
Sports and recreation			
Activity spaces	Sports center; Sports ground; Stadium; Swimming pool	SMO	**13.3**
Children playground	Playground	LCS	
Nature			
Water features	Fountain; Pond; Stream	SMO	**66.0**
Wildlife and Gardens	Wildlife; Gardens		
Trees	NDVI	SPOT-5	
Walking Path	Footpath; Pavement; Pavement under other structure	SMO	
General amenities			
Public art	Declared monument; Church; Municipal services building; Government offices; Library; Mosque; Museum; Pavilion; Temple; Theatre; Tsz Tong	SMO	**20.7**
Public toilets	Public Toilets	SMO	
Car parking	Car park; MTR access; Bus terminus; Minibus terminus	SMO	
Barbeque facilities	Barbeque site	LCS	

Significant are in value [bold].

The three categories (sub-divided into 10 attributes) are adopted from the Desktop Auditing Public Open Space Tool Survey. Weights for each category is re-organized based on the importance of each attribute to participation in physical activity. Data sources are also indicated, in which SMO is the Mapping Offices of Land Department in Hong Kong, LCS is the Leisure and Cultural Services Department in Hong Kong. NDVI data are from SPOT-5 with 10-m resolution for the year of 2008, which should be representative for the current spatial vegetation coverage since there was no significant vegetation change in downtown Hong Kong^48^.

### Modeling accessibility to urban parks

#### Resident-park interaction models

Evidence from previous studies have suggested that urban residents prefer closer, larger, and more attractive urban open spaces^3,14^, which were found to be more strongly associated with adult residents’ recreational walking^15^. To quantify the contributions of different factors in modeling geographic accessibility of urban parks by residents, resident-park interaction models have been defined based on the following assumptions: (1) the interaction declines as the travel distance (or time) between a residential place and a destination park increases, and (2) the interaction improves as the supply capacity and quality of a destination park improves. The quality of park defines the attractiveness of a park for residents by, such as, the facilities and amenities in a park^14,42^. Originated from the gravitational model, the resident-park interaction model can be defined using (1) park size, (2) travel distance (or time) between parks and residents, and (3) attractiveness of the park, with different decay parameters for each of the three factors^16^. The gravity model, a commonly employed spatial interaction modeling technique, gauges the potential accessibility of parks in terms of spatial distribution^8,9,14,47,49^. This method has been broadly adopted in the literature for assessing the accessibility of neighborhood parks. It operates on the premise that as the spatial separation (whether in terms of travel distance or time) between starting points and destinations grows larger, the spatial interaction diminishes. Conversely, spatial interaction escalates when there is greater demand at the starting points or when the destinations boast increased capacity and/or attractiveness. Consequently, residential areas located closer to parks are expected to exhibit enhanced potential park accessibility, just as larger parks are predicted to draw in a greater number of residents. The interaction model used in this study from residential unit $i$ to park $j$ is given by,

2 $$A_{p,ij} = \frac{{attr_{j}^{\lambda } S_{j}^{\alpha } }}{{p_{ij}^{\beta } }}$$

where $p$ is a measure of proximity by either travel distance or time; ${S}_{j}^{\alpha }$ is the size of park $j$ with size-decay parameter $\alpha$, ${p}_{ij}^{\beta }$ is the travel distance or time from residential unit $i$ to park $j$ with distance-decay parameter $\beta$, and ${attr}_{j}^{\lambda }$ is attractiveness of park $j$ with attraction-decay parameter $\lambda$. We use the same decay parameter for travel distance and time given their similar physical meanings in measuring proximity to an urban park. To quantitatively estimate these decay-parameters, a comprehensive survey is required to investigate the correlation between the opportunities in accessing urban park facilities and the three factors. However, such statistics are currently not available in Hong Kong. Instead, we adopted the results from Giles-Corti et al.^14^, which estimated the decay parameters for distance (1.91), attractiveness (0.52), and park size (0.85) based on the data collected from a social ecological project on environmental determinants of physical activity. Using the accessibility models developed in this study, future estimates of the parameters can be readily incorporated when they are available. Note that the main purpose of this study is to develop an urban park accessibility modeling approach that is flexible and can be universally adopted in all high-density cities. Changes of input parameters that are locally suitable are expected when this developed approach is applied to other cities.

#### Population weighted proximity

The population-weighted distance (PWD), a measure of residential population’s spatial access to parks, provides an accessibility metric weighted by population distribution and has shown its capability and effectiveness in quantifying park accessibility in the US^10^. PWD is advantageous since it combines the advantage of classic park accessibility models with information processing theory and probability access surface model. In this study, based on the concept of PWD, we develop a universal framework, population-weighted proximity (PWP). Compared to PWD, PWP aims to have a more comprehensive modeling of urban park accessibility by improving in the following three aspects: (1) using the route distance which is more realistic than traditional geometric distance; (2) incorporating park attractiveness quantified using park attributes, facilities, and amenities; (3) using travel time instead of travel distance to measure spatial proximity.

After modeling the resident-park interaction, $A$, as shown in Eq. (2), the probability (${P}_{p,ij}$) of a person living in resident $i$ to visit park $j$ can be quantified by the ratio of potential accessibility to neighboring urban parks, given by,

3 $$P_{p,ij} = \frac{{A_{p,ij} }}{{A_{p,i} }};{ }\;\;{ }where{ }A_{p,i} = { }\mathop \sum \nolimits_{j = 1\sim k} A_{p,ij}$$

where $k$ is the number of neighboring urban parks, $p$ is a measure of proximity by either travel-distance or -time. $k$ is set to be 7, an estimate by Zhang et al.^10^ based on the information processing theory, which suggested that the seven nearest parks can be the most probable choice set that an urban resident takes into consideration when deciding a preference park to visit. Finally, PWP for each SB level planning unit is defined as the expected distance or time for a SB unit ($i$) with population (${Pop}_{i}$) to its nearby urban parks, which is given by,

4 $$PWP_{p,i}^{sb} = { }\mathop \sum \nolimits_{j = 1\sim k} \left( {Pop_{i} {*}P_{p,ij} {*}p_{ij} } \right)/Pop_{i}$$

Similarly, the PWP for larger planning units, such as TPU, DC or even whole Hong Kong territory, to visit the nearest urban parks can be calculated by aggregating the results at the SB levels weighted by population, which is given by,

5 $${\text{PWP}}_{{{\text{p}},{\text{i}}}}^{{\text{x}}} = { }\mathop \sum \nolimits_{{{\text{i}} = 1\sim {\text{n}}}} \left( {{\text{Pop}}_{{\text{i}}} {\text{*PWP}}_{{{\text{p}},{\text{i}}}}^{{{\text{sb}}}} } \right)/{\text{Pop}}_{{\text{x}}}$$

where $\mathrm{n}$ is the number of SBs in a larger planning unit $\mathrm{x}$, which can be TPU, DC or the whole Hong Kong territory, and ${\mathrm{Pop}}_{\mathrm{x}}$ is the total population of the planning unit $\mathrm{x}$.

#### Comparisons of resident-park interaction models

One of the advantages of the resident-park interactions model is its flexibility to incorporate different factors that have an impact on urban park accessibility. By constructing different resident-park interaction models, we can quantify the impact of different factors in measuring park accessibility. In this study, the following four models are compared to investigate (1) the difference between using geometric and route distance, (2) the effect of park attractiveness in measuring urban park accessibility, and (3) the difference between using travel-time and -distance to measure spatial proximity between residents and urban parks.

**Model 1**. Population-Weighted Geometric Distance (PWGD) to urban parks. The interaction model is defined by,

6 $${\text{A}}_{{{\text{d}},{\text{ij}}}}^{1} = \frac{{{\text{S}}_{{\text{j}}}^{{\upalpha }} }}{{{\text{d}}_{{{\text{g}},{\text{ij}}}}^{{\upbeta }} }}$$

in which only the park area (${\mathrm{S}}_{\mathrm{j}}^{\mathrm{\alpha }}$) and geometric distance (${\mathrm{d}}_{\mathrm{g},\mathrm{ij}}^{\upbeta }$) are considered.

**Model 2**. Population-Weighted Route Distance (PWRD) to urban parks. The interaction model is given by,

7 $${\text{A}}_{{{\text{d}},{\text{ij}}}}^{2} = \frac{{{\text{S}}_{{\text{j}}}^{{\upalpha }} }}{{{\text{d}}_{{{\text{r}},{\text{ij}}}}^{{\upbeta }} }}$$

in which only the park area (${\mathrm{S}}_{\mathrm{j}}^{\mathrm{\alpha }}$) and route distance (${\mathrm{d}}_{\mathrm{r},\mathrm{ij}}^{\upbeta }$) are considered.

**Model 3**. Population-Weighted Route Distance with Attractiveness (PWRD-Attr) to urban parks. The interaction model is given by,

8 $${\text{A}}_{{{\text{d}},{\text{ij}}}}^{3} = \frac{{{\text{attr}}_{{\text{j}}}^{{\uplambda }} {\text{S}}_{{\text{j}}}^{{\upalpha }} }}{{{\text{d}}_{{{\text{r}},{\text{ij}}}}^{{\upbeta }} }}$$

in which the park attractiveness index (${\mathrm{attr}}_{\mathrm{j}}^{\uplambda }$) is added to model 2.

**Figure 4:**
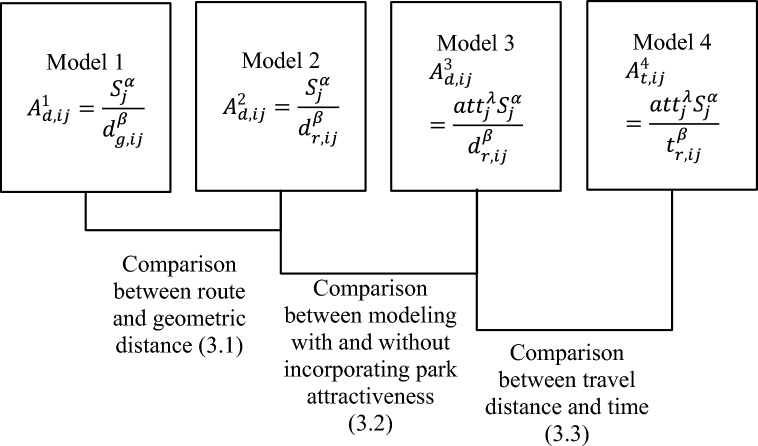
Schematic work flow in this study. The four resident-park interaction models for measuring urban park accessibility are shown in rectangles and three comparison studies based on these models are also indicated with their section numbers shown in parenthesis.

**Model 4**. Population-Weighted Route Time with Attractiveness (PWRT-Attr) to urban parks. The interaction model is given by,

9 $${\text{A}}_{{{\text{t}},{\text{ij}}}}^{4} = \frac{{{\text{attr}}_{{\text{j}}}^{{\uplambda }} {\text{S}}_{{\text{j}}}^{{\upalpha }} }}{{{\text{t}}_{{{\text{r}},{\text{ij}}}}^{{\upbeta }} }}$$

in which the travel route time, instead of distance, is considered compared to model 3. The structure of these four models and the comparisons between them are shown in Fig. 4.

The calculation of the urban park accessibility for further analysis can be summarized as follows. Firstly, the resident-park interaction metric, ${\mathbf{A}}_{{\varvec{d}},{\varvec{i}}{\varvec{j}}}$ is calculated for every street block to the existing parks following Eqs. (6), (7), (8), or (9) depending on the model assumption; Secondly, we calculated the probability (${{\varvec{P}}}_{{\varvec{p}},{\varvec{i}}{\varvec{j}}}$) of a person living in resident ${\varvec{i}}$ to visit park ${\varvec{j}}$ following Eq. (3). The total number of neighboring parks to be considered is ${\varvec{k}}$, which is set to be 7. ${\varvec{p}}$ is a measure of proximity by either travel-distance or -time.; Lastly, PWP for each SB level planning unit is defined as the expected distance or time for a SB unit (${\varvec{i}}$) with population (${{\varvec{P}}{\varvec{o}}{\varvec{p}}}_{{\varvec{i}}}$) to its neighboring urban parks, as shown in Eqs. (4) and (5). PWP is the accessibility shown in the following result section.

## Results

Urban park accessibility for the high-density urban areas of Hong Kong are quantified in this study using the developed population-weighted proximity model. The urban park accessibility is calculated using different resident-park interaction models. A comparison of different measures of resident-park interaction models are conducted to assess the contributions from difference factors, including walking distance (geometric and route distances), park size and attractiveness, and walking time. In particular, the strengths of using walking time and distance predicted by Google Maps crowdsourcing database and incorporating park attractiveness index are highlighted.

### Modeling urban park accessibilities with *geometric and route distances*

**Figure 5:**
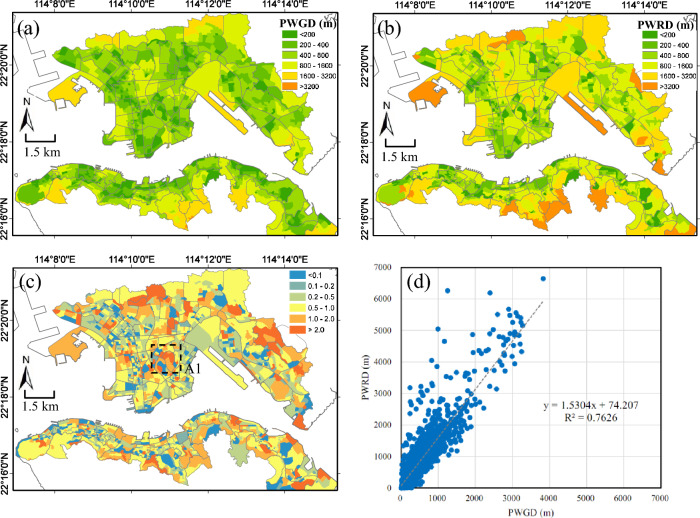
(**a**) Street block (SB) level of population-weighted geometric distance (PWGD) of urban parks accessibility, and (**b**) SB level of population-weighted route distance (PWRD) of urban parks accessibility in high density urban areas of Hong Kong; (**c**) The relative difference, (PWRD-PWGD)/PWGD, between PWGD and PWRD of urban parks accessibility for SB planning units. The black rectangles (labeled by A1) in (c) is a hot spot with high population density as well as larger difference in accessibility; **(d)** Scatter plot and linear regression (both equation and R^2^ are indicated) of PWRD and PWGD for all SB planning units in high density urban areas of Hong Kong. The maps are created using ArcGIS software (version 10; https://www.esri.com/).

The Fig. 5a, b depict the PWGD (Eq. (6)) and PWRD (Eq. (7)), respectively, of urban parks accessibility at SB level, and their difference in (c). We can see both PWGD and PWRD show similar patterns, with lower values (or better accessibility) in areas with denser urban parks, as shown in Fig. 2a. Generally, SBs with the highest population density (Fig. 1) as well as urban parks density (Fig. 2a), such as the large metropolitan and highly urbanized neighborhoods, have the shortest distance (or best accessibility) to urban parks. This suggests that the density of urban parks is positively related to accessibility to urban parks. Moreover, we can see that the PWRD value is generally larger than PWGD value because the route distance is always equal or larger than geometric distance, as shown in Fig. 3a. On average, residences in this high-density urban area of Hong Kong are expected to walk 598 m by PWGD metric and 990 m by PWRD metric to access their local neighborhood urban parks.

The relative differences, (PWRD-PWGD)/PWGD, as shown in Fig. 5c, are non-uniformly distributed across the study area, with higher difference mostly closes to the edges of the study areas where population density (Fig. 1) is generally lower and streets network is less dense (Fig. 2a). We can see that most of these areas have sparse streets which make the route longer to walk to urban parks while the geometric distance is comparatively much shorter. Since route distance is a measure based on the street network while geometric distance based on the straight geometric line, their difference can be an indicator to assess the effectiveness of the local street networks. For example, the black rectangles in (c) is one of the hot spots with high population density as well as the larger difference in accessibility, indicating that in this neighborhood more streets are in need to improve the accessibility to local neighborhood urban parks. A comparison between PWGD and PWRD show that these two measures are strongly correlated (r^2^ = 0.76), especially in large metropolitan areas (where there are denser street networks and urban parks and therefore the distances are relatively smaller) as shown in Fig. 5d. Similar to Fig. 3b, PWRD and PWGD have a conversion factor of about 1.53. These results indicate that the PWRD measure avoids the potential bias associated with the geometric distance measure in PWGD (e.g., Zhang et al.^10^) and provides a more realistic picture of potential spatial access of residential population to urban parks.

### Modeling urban park accessibilities with *park attractiveness index*

**Figure 6:**
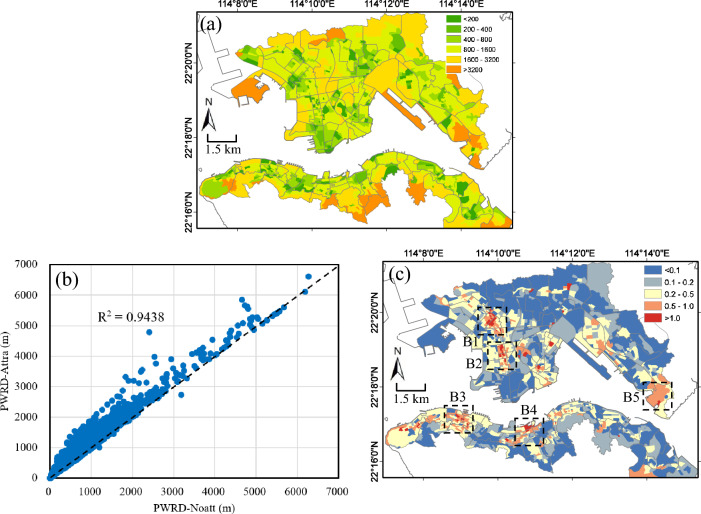
(**a**) Street block (SB) level of population-weighted route distance with attractiveness (PWRD-Attr) of urban park accessibility in density urban areas of Hong Kong; (**b**) Scatter plot of PWRD and PWRD-Attr of urban park accessibility (R^2^ from correlation analysis is also indicated). The dashed line is the 1:1 line; (**c**) Relative difference, (PWRD-Attr – PWRD)/PWRD, between PWRD and PWRD-Attr. The black rectangles (labeled by B1 to B5) in (**c**) are hot spot regions with high population density as well as larger difference in accessibility. The maps are created using ArcGIS software (version 10; https://www.esri.com/).

The Fig. 6a shows PWRD-Attr (Eq. (8)) of urban parks accessibility at the SB level. In general, we can see both PWRD and PWRD-Attr show similar patterns that neighborhood areas closer to denser urban parks have better accessibility. The scatter plot of PWRD and PWRD-Attr in Fig. 6b shows a good correlation between the two measurements with R^2^ of 0.94. However, we can see that PWRD-Attr data are generally larger than PWRD. This is because, when considering the attractiveness, urban residents are more willing to walk to farther parks with higher attractiveness which have larger area or more facilities. Interestingly, we can see that when the distance is over 5000 m, the PWRD and PWRD-Attr values are almost the same, suggesting that this attractiveness factor has little effect for residents to visit an urban park. This may indicate that when the walking distance is over 5000 m, the importance of distance outweighs park attractiveness in accessing urban parks. The mean value of walking distance a resident expected to walk is 1210 m when park attractiveness is considered, which is longer than the mean value of PWRD without attractiveness, i.e. 990 m, by about 22%.

The Fig. 6c shows the spatial difference between PWRD and PWRD-Attr. Similar to Fig. 6b, we can see that PWRD-Attr is generally larger than PWRD, with the largest difference mainly distributed in areas, labeled as B1 to B5, where there are denser street networks but the surrounding parks in these areas have very limited facilities and amenities inside. Therefore, when park attractiveness is considered in modeling the resident-park interactions, our model clearly captures the realistic situation that urban residents tend to travel longer distance to parks with more attractiveness. Especially for areas B1 and B2, there are high-density population up to 100,000 person / km^2^ but the neighborhood parks have very limited facilities and amenities, suggesting that these communities have a pressing need to increase the number of new parks or add facilities to improve the attractiveness of existing neighborhood parks. For areas where the difference is very small, that is, the park's attraction model has no significant impact, it indicates that the parks in these areas have relatively good quality of provision.

### Modeling urban park accessibilities with *travel time*

**Figure 7:**
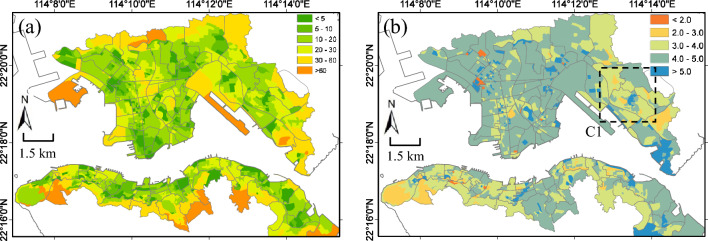
(**a**) Street block (SB) level of population-weighted route time with park attractiveness considered (PWRT-Attr) of urban park accessibility in high density urban area of Hong Kong; (**b**) Averaged walking speed (km/hour), calculated by dividing PWRD-Attra by PWRT-Attra, which can be influenced by different street conditions, such as street slopes and traffics, in accessing urban parks. The black rectangle (labeled by C1) in (**b**) is one hot spot region with high population density as well as smaller walking speed in accessibility to neighborhood urban parks. The maps are created using ArcGIS software (version 10; https://www.esri.com/).

Walking time, compared to walking distance, is a more effective indicator of accessibility to urban parks since walking time is a direct and human-based measure of efforts in accessing urban parks. It can be influenced by distance as well as other factors including street conditions such as street slopes and traffic. The walking time and distance should show the same pattern if the walking speed is constant. However, in reality, walking speeds are affected by different street conditions, especially in Hong Kong Island where there are varying street elevations and in Kowloon where there are heavy traffic. Based on historical walking and traffic condition data collected by Google Maps^40,43^, the optimal route and best estimate of walking time from Google GMDM provide a new data source to investigate the accessibility in terms of walking time. Figure 7a shows the walking time (PWRT-Attr from Eq. (9)) that residents in each SB used to visit neighborhood parks. The averaged walking time over the whole study area is 18 min. Generally, the pattern of walking time is consistent with walking distance as Fig. 6a. Some areas which close to urban parks have walking time within 10 min, while other areas further away may need to walk for more than 30 min to access neighborhood urban parks.

To investigate the impact of street conditions on walking accessibility, Fig. 7b shows the walking speed calculated by dividing PWRD-Attr (Fig. 6a) by PWRT-Attr (Fig. 7a). We can see the walking speed for most of the study areas is between 4.0–5.0 km/hour (that is, 1.1–1.4 m/second), which is close to the preferred walking speed for human (5.0 km/hour; ref.^50^). However, some areas closing to the southern Hong Kong Island and eastern Kowloon where the routes have higher slopes tend to have a slower walking speed less than 4.0 km/hour. For urban residents in these areas, alternative travel method, such as public transportation instead of walking, should be provided by public service.

### Modeling urban park accessibilities at TPU level

**Figure 8:**
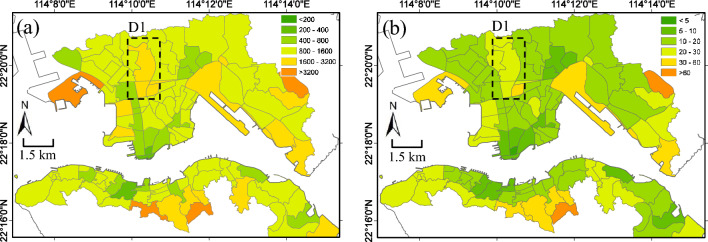
Urban park accessibility of (**a**) Population-weighted route distance with attractiveness (PWRD-Attr), and (**b**) Population-weighted route time with attractiveness (PWRT-Attr) at TPU planning unit level, aggregated from SB level of accessibility. The area D1 (highlighted in dashed box) is located in central Kowloon with dense population but has a relatively lower accessibility in both PWRD-Attr and PWRT-Attr. The maps are created using ArcGIS software (version 10; https://www.esri.com/).

One of the advantages of the population-weighted accessibility modeling approach is that when aggregating to larger planning unit (such as TPU) from the weighted average of basic units (such as SB), weights quantified by population densities accounts the homogeneous population distribution which makes the accessibility metric more of a human-based indicator. TPUs in Hong Kong are demarcated for the purpose of town planning by the Planning Department of the Government. An assessment of accessibility to urban parks at TPU level can be directly linked to urban planning policies in this high-density urban area of Hong Kong. Figure 8 shows the aggregated PWRD-Attr and PWRT-Attr at TPU level. Both metrics show a similar pattern with better accessibility in areas generally with denser urban parks (see Fig. 2a) and population (see Fig. 1). Urban residents in most of the TPUs have access to neighborhood parks within a walking distance of 1.6 km and walking time of 20 min. The area (highlighted in dashed box) is located in central Kowloon with dense population but has a relatively lower accessibility in both walking distance and time. By comparing the park density in Fig. 2a, we find the TPUs in this area has sparser urban parks than surrounding regions, suggesting that more urban parks need to be built in this area to improve accessibility.

## Discussion

### Strength of using Google Maps crowdsourcing database for accessibility modeling

The Distance Matrix API by Google Maps uses crowd sourced data and offer a quick and accurate way of solving the shortest path problem for modeling accessibility. Compared to conventional method that relies on GIS spatial analysis, using Google Maps API comes with a frequently updated road network dataset that users do not need to prepare by themselves. What is more important is that the crowd sourced data in Google Maps API contain abundant information about road and traffic conditions that vary by time of the day, which are sometimes hard to collect for individual researcher. Overall, Google Maps API makes it possible to assess urban park accessibility in this study in a more accurate and realistic way.

### Contribution of park attractiveness and walking time in accessibility modeling

In this paper, four models of resident-park interactions (Eq. (6)–(9)) are developed to assess the accessibility of urban parks in high-density urban areas of Hong Kong. As a result, residents in the study area are, on average, expected to walk for 598 m (PWGD), 990 m (PWRD), and 1210 m (PWRD-Attr) or 18 min (PWRT-Attr) to visit the neighborhood urban parks. A comparison of the four models is shown in Fig. 8 We can see that a majority of urban parks are within walking distance of 1000 m (by PWRD-Attr) or 20 min (by PWRT-Attr). By adding parameters in the interaction model from PWGD to PWRD and to PWRD-Attr, we find the distribution of walking distance shifts to a higher value, showing that a long distance to access the neighborhood urban parks. On average, the route distance is longer than the geometric distance by about 1.5 times. When taking attractiveness into account, the expected walking distance is increased by about 1/3. These significant differences suggest that the design of street networks to urban parks might need to be revisited in Hong Kong and urban parks closer to areas with denser population should improve their attractiveness. The contribution of different factors in modeling resident-park interactions can be concluded as follows: (1) Park attractiveness. This study revisits the common assumption that park size can be a proxy for quantifying its attractiveness and showed that (a) the correlation between park size and its attractiveness has large uncertainty, and (b) when park attractiveness is considered in modeling the resident-park interaction, urban residents in high-density urban areas of Hong Kong tend to travel longer distance (on average longer by about 1/3) to urban parks with more attractiveness. Therefore, the common assumption on park size and its attractiveness may not necessarily feasible, and park attractiveness should be considered in order to have a more reasonable and effective modeling of resident-park interactions. (2) Walking time. Walking time is a direct and human-based measure of efforts in accessing urban parks. It can be influenced by distance as well as other factors including street conditions such as street slopes and traffic. Our results show that the walking time, quantified by PWRT-Attr, provides us an extra dimention to investigate the urban park accessibility from the perspective of street conditions, and therefore offer some conceptual improvement upon the simpler metrics by traditional distance.

### Implications on building a walkable Hong Kong

Results from this study have important policy implications on urban park planning and design for developing a healthy living environment in Hong Kong. To promote Hong Kong as a walkable city with healthy and environment-friendly living styles, city planner should pay attention to areas without good access to urban green spaces when planning new parks or improve existing parks. From this study, several different regions short of park accessibility or park attractiveness have been detected and highlighted by comparing different resident-park interaction models. These regions include (1) A1 in Fig. 5 where denser street networks that connecting neighborhood parks are needed, (2) B1-5 in Fig. 6 where park attractiveness needs to be improved by introducing more amenities and facilities, (3) C1 in Fig. 7 where street conditions may not be ideal for walking and other alternatives need to be introduced, and (4) D1 in Fig. 8 where urban parks are sparser than other regions and new ones need to be established or existing ones expanded. The Hong Kong Planning Department published a Hong Kong Planning Standards and Guidelines (HKPSG), which suggests that local urban open spaces be located within a short walking distance from the residents it intends to serve, preferably within a radius of not more than about 500 m. From our results on accessibility to urban parks, the average walking distance is over one kilometer on average (by PWRD-Attr), suggesting that there is still a large gap in improving accessibility in this high-density urban area of Hong Kong.

## Conclusions

In this paper, we propose an novel approach that makes use of Google Maps crowdsourcing database and incorporates the park attractiveness to model the urban park accessibility. The effectiveness of the developed approach is illustrated by applying it to the complex high-density urban regions in Hong Kong. Through inter-comparisons of different accessibility models from simplified to comprehensive settings, we quantify the impact of different factors in measuring urban park accessibility, including (1) the difference between using geometric and route distance, (2) the effect of park attractiveness in measuring urban park accessibility, and (3) the difference between using walking time and distance in measuring spatial proximity between residents and urban parks.

The results show that the residents in high-density urban areas of Hong Kong are, on average, expected to walk for 1200 m and 18 min, respectively, to access the neighborhood urban park. We found that the route and geometric distances have a strong correlation with a conversion factor of about 1.5. The results also suggest that park attractiveness should be explicitly considered for a more effective accessibility modeling and cannot be assumed to be proportional to park size. Moreover, walking time to access urban parks shows that there are non-negligible impacts from street conditions and traffic on urban park accessibility. Lastly, several community hotspots in Kowloon and Hong Kong Islands short of park accessibility or attractiveness have been explicitly detected and highlighted.

The developed approach in this study has a number of strengths, including (1) the population-weighted models are flexible and informative in characterizing the interactions between urban residents and urban parks, (2) the optimal walking distance/time estimated by Google Maps API based on the historical walking and traffic condition data provides a more realistic quantification of accessibility in urban areas, and (3) the differences between the four resident-park interaction models provide unique ways for city planners to define the contributions of street networks, park size and attractiveness, and street conditions, and understand which of them should be prioritized to improve accessibility at different areas. The developed approach provides a flexible and informative tool for city planners, health professionals, and policy makers to evaluate and improve greening planning and design in high-density cities.

For future studies, more factors need to be incorporated in the urban park accessibility modelling. For example, while travel distance/time and destination attractiveness contribute to accessibility modeling, the experience during travel is non-negligible. For instance, the presence of funeral parlors or trash recycling stations along the way may reduce accessibility, especially in Hong Kong, known for its mixed-use land characteristics. Theoretically, different attractiveness weights should be assigned to different land uses. It is therefore important to investigate the role of route attractiveness when modelling walking accessibility to urban parks in Hong Kong.

In addition, improvement can be made by resolving the accessibility heterogeneity at street and building level within each SB unit. The attractiveness index is a standard calculation for urban parks with different characteristics and residents with all age groups. It is possible to adjust the weights for facilities and amenities according to different type/theme of urban parks in Hong Kong based on more surveys from future studies. Moreover, the weights for a certain urban park may be different for different age group, such as young people would more prefer urban parks with sports facility while elderly prefer urban parks with more greenery and walking trails. Also, this study focuses on urban parks, an important type of green spaces in Hong Kong, which is more accessible by residents in daily life. Accessibility to country parks, another important green space which attracts residents to visit especially over the weekend, may be investigated in future studies. Lastly, a comprehensive assessment of street walkability by combining urban-park accessibility with street-level greening^51,52^ and solar exposure^53^ will be another important research topic toward building a walkable Hong Kong.

## Data Availability

The datasets generated and analysed during the current study are available in the Zenodo repository at https://doi.org/10.5281/zenodo.8169274.
